# Short-term detection of volcanic unrest at Mt. Etna by means of a multi-station warning system

**DOI:** 10.1038/s41598-019-42930-3

**Published:** 2019-04-24

**Authors:** Salvatore Spampinato, Horst Langer, Alfio Messina, Susanna Falsaperla

**Affiliations:** 1 Istituto Nazionale di Geofisica e Vulcanologia, Sezione di Catania, Osservatorio Etneo, Piazza Roma 2, 95125 Catania, Italy; 2 Istituto Nazionale di Geofisica e Vulcanologia, Sezione Roma2, Via di Vigna Murata 605, 00143 Roma, Italy

**Keywords:** Volcanology, Natural hazards

## Abstract

Early-warning assessment of a volcanic unrest requires that accurate information from monitoring is continuously gathered before volcanic activity starts. Seismic data are an optimal source of such information, overcoming safety problems due to dangerous conditions for field surveys or cloud cover that may hinder visibility. We designed a multi-station warning system based on the classification of patterns of the background seismic radiation, so-called volcanic tremor, by using Self-Organizing Maps (SOM) and fuzzy clustering. The classifier automatically detects patterns that are typical footprints of volcanic unrest. The issuance of the SOM colors on DEM allows their geographical visualization according to the stations of detection; this spatial location makes it possible to infer areas potentially impacted by eruptive phenomena. Tested at Mt. Etna (Italy), the classifier forecasted in hindsight patterns associated with fast-rising magma (typical of lava fountains) as well as a relatively long lead time of the outburst (lava flows from eruptive fractures). Receiver Operating Characteristics (ROC) curves gave an Area Under the Curve (AUC) ∼0.8 indicative of a good detection accuracy that cannot be achieved from a mere random choice.

## Introduction

After the UN proclaimed the 1990’s as the International Decade for Natural Disaster Reduction, the need for more resilient communities to natural disasters has greatly increased^1^. The Indian Ocean tsunami in 2004^2^ and the 2011 earthquake in Japan^3^ have further fostered worldwide attention to the importance of prevention and mitigation of natural disasters promoting effective early warning systems, from the improvement of real-time evaluation of data from monitoring to the communication to at-risk populations.

Among the four fundamental elements of an early warning system – (1) risk knowledge; (2) monitoring and warning service; (3) dissemination and communication; (4) response capability – the development of efficacious hazard monitoring entails the identification of parameters that allow forecasts on a sound scientific basis, providing timely warnings^4^. In a volcanic environment, a key issue is the timely detection of anomalies in the monitored signals that may herald an eruptive event with potentially dangerous impact. To contribute to early warning services, volcano observatories systematically collect and analyze data, implementing advanced tools for online and near-real time data processing^5^. Automated data processing has been proposed for the monitoring of several volcanoes, such as Kilauea^6^ (Hawaii), Soufrière Hills volcano^7^ (Montserrat), and Piton de la Fournaise^8^ (Réunion).

Mt. Etna, in Italy, is the largest and most active volcano in Europe; it dominates the topography of the metropolitan area of Catania, with almost a million people living on its flanks and thousands of tourists visiting its summit area every year. Considering also the important infrastructures built in this area, such as the International Airport Fontanarossa, as well as the Catania - Messina and Catania - Palermo freeways, the early detection of volcanic unrests is a key issue for risk mitigation and Civil Protection purposes. Etna offers a good example of a developed hazard monitoring^9^. Here besides field surveys, the monitoring system encompasses gas measurements and images from video-cameras along with the continuous record of geophysical data streams. Among the latter, seismic signals play a key role for early warning purposes, as they provide timely, valuable insights into the processes leading to magma uprising and eruptive activity^10^. Seismic signals come from a dense network of permanent three-component broadband seismic stations, and allow us continuous remote control on the state of the volcano, overcoming safety problems for field surveys or missing information from video and satellite monitoring due to bad-weather conditions. In this framework, we designed a multi-station warning system based on pattern recognition to detect the volcano unrest from volcanic tremor, the background seismic radiation^11^. We present the system performance for the hindsight forecast of the eruptive episodes in 2011- encompassing 18 lava fountains - and the 2008–2009 flank eruption.

### Etna: Scenarios of volcanic activity and recent eruptive centers

**Figure 1:**
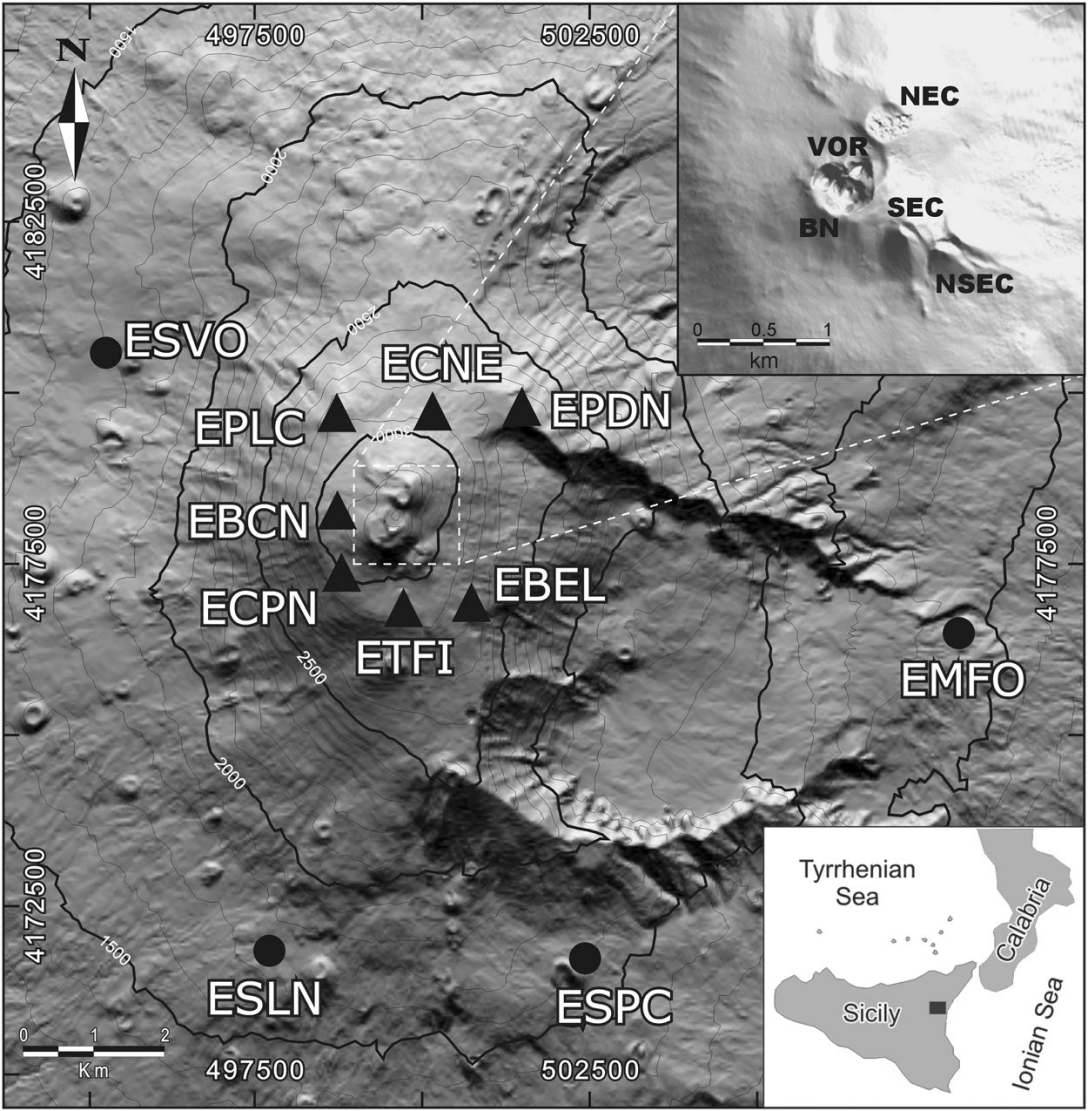
DEM of Etna and sketch map of the geographical position (bottom inset on the right). Triangles and circles mark the location of the permanent seismic stations we used in this study, which approximately form two rings with center at the summit craters: an inner (within a radius of ∼3 km) and outer (at distance up to ~8 km) ring, respectively. The shaded image of the DEM is from Favalli *et al*.^32^, reprinted by permission of the publisher (Taylor & Francis Ltd, http://www.tandfonline.com). The top inset on the right is a DEM of Etna summit craters in 2012: North East Crater (NEC), Bocca Nuova (BN), Voragine (VOR), South East Crater (SEC), and New South East Crater (NSEC). This DEM was realized by the “Laboratorio di Aerogeofisica” of INGV Sezione Roma 2 exploiting aerial images taken during a helicopter survey and processed by “Agisoft Photoscan Pro v.1.2”, a software for SfM photogrammetry^33^ (http://geodb.ct.ingv.it). Geographical coordinates of both DEMS are expressed in UTM projection, zone 33 N.

The first eruptive activity at Etna dates back to about 600 ka ago and set up the basaltic stratovolcano in a complex geodynamic environment between the Gela-Catania foredeep and the Hyblean Foreland, which is the front of the orogenic belt overlapping the African continental plate margin^12^. North East Crater (NEC), Bocca Nuova (BN), Voragine (VOR), South East Crater (SEC), and New South East Crater (NSEC) are the present main eruptive centers located at the top of the volcano, which is 3329 m high^13^ (Fig. 1). Etna had two main eruptive styles in the last two decades: (i) lava fountains, and (ii) lava effusions. Lava fountains stem from the summit craters tens of kilometers distant from urban areas. Although they are not a direct menace for the local population, lava fountains often gush out dense ash clouds in the atmosphere, forming a volcanic plume taken (even hundreds of kilometers) away by winds^14^. The consequent ash fallout may yield problems to agriculture, human and livestock health as well as disrupt road and air traffic. Compared to lava fountaining, which is a short-lived (a few hours-long) phenomenon, a lava effusion may last several months or even years (for example, the 2008–2009 lava eruption lasted 419 days^15^). Therefore, it is potentially more dangerous, especially in case of flank eruptions, during which the lava emission comes from eruptive fractures at low altitude. Consequently, lava effusions are a potential threat for the densely populated urban areas located along the flanks of the volcano. Devastating effects due to lava-flow invasions are documented for the lava effusions in 1669^16^ and 1928^17^.

### Data Analysis

Volcanic tremor is a continuous signal at Etna owing to the persistent movement of fluids within the volcano^11^. Several studies have documented that its amplitude and frequency content have a strict link with changes in the internal dynamics of Etna’s volcano feeder^18,19^ (see Supplementary Information). For early-warning purposes, volcanic tremor analysis brings along the necessity of continuous acquisition and automatic data processing, making data reduction and parameter extraction a primary task to cope with. Note that for one seismic station with sampling rate of 100 Hz it is required the storage of ∼12 GB of binary data per channel per year. Langer *et al*.^20^ proved that pattern classification – in particular Self-Organizing Maps, henceforth SOM^21^ – provides not only a strong data reduction, but also allows the efficient detection of a volcanic unrest from the spectral characteristics of the seismic signal. Pattern recognition is actually implemented in many classification problems for monitoring purposes: SOM were applied to investigate the relationship between the occurrence of “Very Long Period” events recorded at Stromboli volcano, Italy, and variations in infrared images at the active craters^22^; the classification of seismic signals based on Hidden Markov Models was explored in various publications, e. g., Hammer *et al*.^7^ who considered data recorded at the Soufrière Hills volcano, Montserrat; Hibert *et al*.^8^ applied the” Random Forest” technique for the identification of rockfalls and Volcano-Tectonic events at Piton de la Fournaise, Réunion.

We probed SOM in the framework of a multi-station system aimed at early warning. We started considering the continuous data stream from 11 permanent seismic stations. The latter formed two rings with center at the summit craters: an inner ring of seven stations (within a radius of ∼3 km), and an outer ring with four stations at distance up to ~8 km (Fig. 1). They were all equipped with Nanometrics Trillium™ seismometers having a natural period of 40 s. The dynamic range of the digitizer and the sampling rate were 24 bits and 100 Hz, respectively.

**Figure 2:**
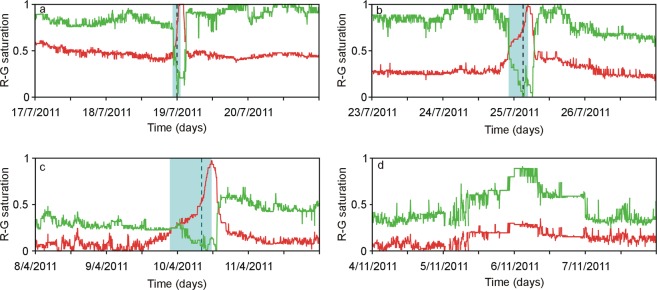
Normalized values of the red (R) and green (G) components in the RGB color code of the SOM results. Note the anti-correlated change of R and G values in case of lava fountain (**a**–**c**). From the cross point of the two lines until the climax of the eruption (light blue rectangle), the increase of R may occur in <3 h (**a**), a few hours (between 3 and 9 h; **b**) or several hours (≥9 h; **c**). After the climax, R drops rapidly and G increases quickly (**a**–**c**). R and G have almost parallel trends during periods of volcano quiescence (**d**). The dashed lines mark the start of lava fountaining (see Table SI2).

**Figure 3:**
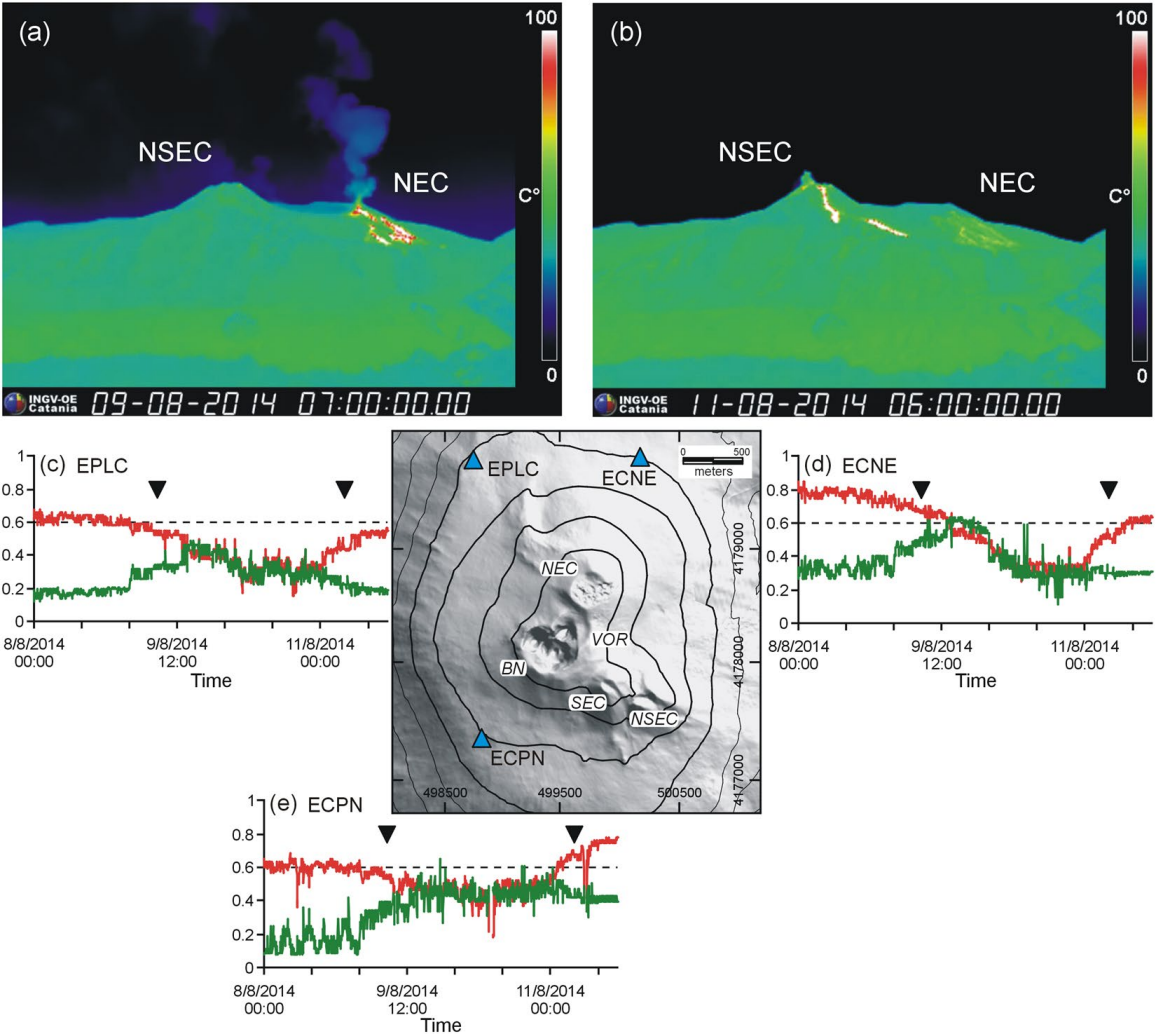
Images of thermal video cameras documenting eruptive activity at NEC (**a**) and NSEC (**b**) on 9 and 11 August 2014, respectively. The DEM (in the middle of the figure) depicts the location of three seismic stations (EPLC, ECNE, and ECPN) and Etna summit craters. Temporal variations of the red and green components of SOM at the three stations are depicted in (**c**–**e**). Black triangles mark the time period of the images shown in (**a**,**b**), respectively. The DEM is owned by INGV (http://geodb.ct.ingv.it); geographical coordinates are expressed in UTM projection, zone 33 N. The DEM was realized by the “Laboratorio di Aerogeofisica” of INGV Sezione Roma 2 exploiting aerial images taken during a helicopter survey and processed by “Agisoft Photoscan Pro v.1.2”, a software for SfM photogrammetry^33^.

Pattern classification was obtained with step of 5 min by using SOM combined with fuzzy clustering, as described in Methodology. The results were expressed as numerical values in the RGB (Red/Green/Blue) color code mirroring the spectral characteristics of the seismic signal. Here we focus on the “red” and “green” components, as the “blue” is inversely related to the green (see Methodology). In particular, before an impending lava fountain, we found the “red” component (R) increased along with a decrease of the green component (G). Connecting the sequential values of these two components with lines in a diagram, we observed that the anti-correlated change of R and G values may occur rapidly (leading from the cross point of the two lines to the eruptive climax in <3 h; Fig. 2a), in a few hours (between 3 and 9 h; Fig. 2b) or slowly (>9 h; Fig. 2c). R and G values for the 18 lava fountaining episodes in 2011 are depicted in Figure SI1. After peaking in the eruptive climax, R dropped rapidly and G increased quickly (Fig. 2a–c). The two lines with R and G values had almost parallel trends during periods of volcano quiescence (Fig. 2d). We also observed that the major increase in the R component marked the station/s closer to the active crater. For example, the summit crater NEC was active during July and in the first half of August 2014. ECNE (the nearest station to NEC) had higher R values than EPLC and ECPN until August 9 (Fig. 3c–e). Between 9 and 11 August, NEC activity declined and the NSEC started erupting (Fig. 3a,b); R values concurrently changed, peaking at the station ECPN, reaching a minimum at EPLC, and showing values at ECNE in between the other two stations (Fig. 3c–e). Therefore, the relative change of the R values among the stations may provide clues about which of the eruptive centers may become active.

**Figure 4:**
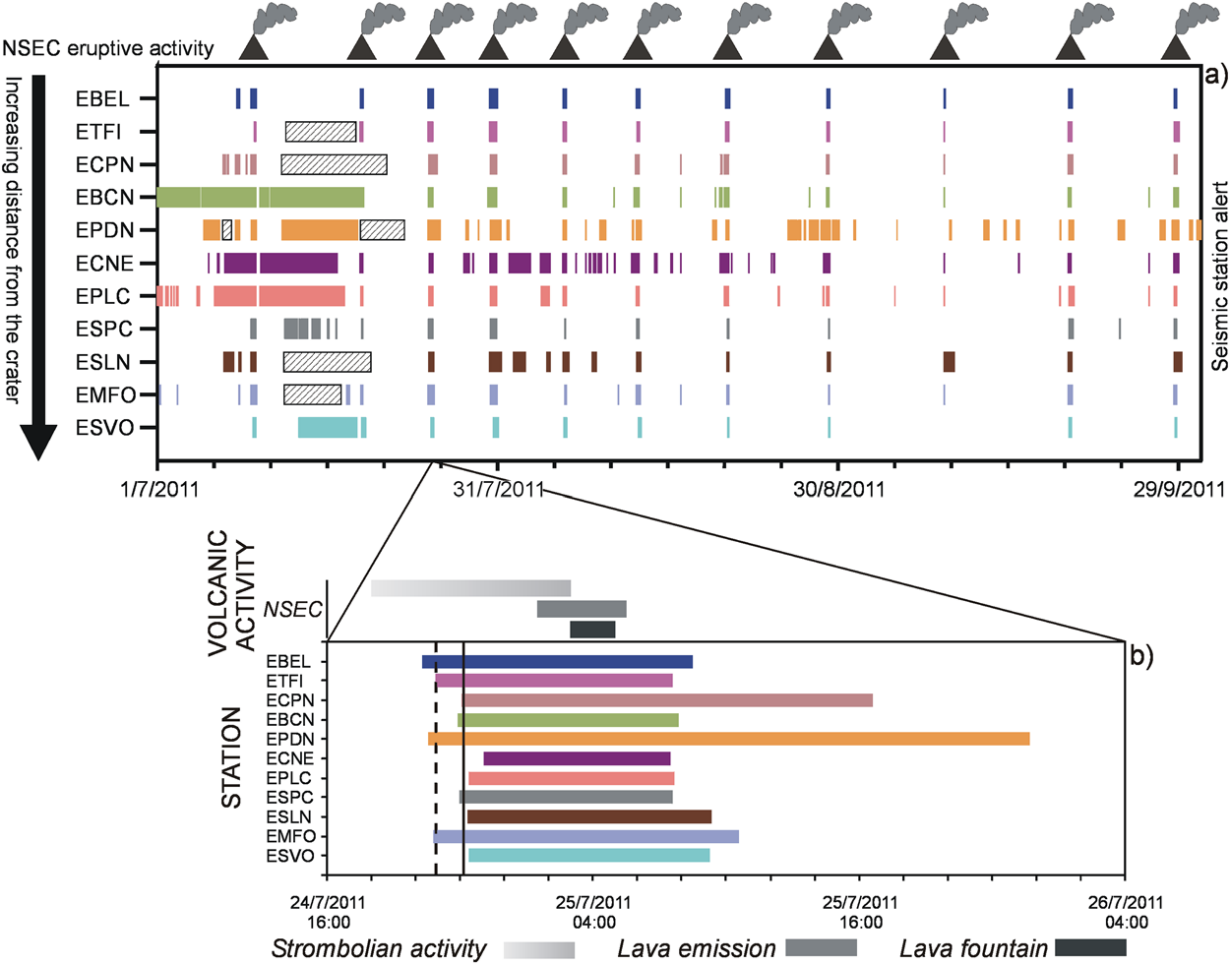
(**a**) Example of warning flag for the lava fountains at Etna between July and September 2011. Lava fountains (here marked by a smoking volcano symbol) stemmed from NSEC. Colored lines depict the duration of the alert at each of the 11 seismic stations (listed in order of increasing distance from the crater). Small hatched boxes mark time spans with uncertain closing time. (**b**) Zoom of the eruptive episode on 25 July; the dashed and solid lines mark the moment when the warning system reaches the second and third alert level, respectively.

Moving on from these observations, we defined trigger parameters for each single station (Table SI1) and probed the classifier using past data streams, encompassing episodes of eruptive activity. Note that the changes in R and G do not allow us any inference into the type of eruptive style. Nevertheless, the example of Fig. 4 depicts that each of the 11 seismic stations was able to issue a timely warning of impeding volcanic activity.

### Multi-station warning system and voting scheme

**Figure 5:**
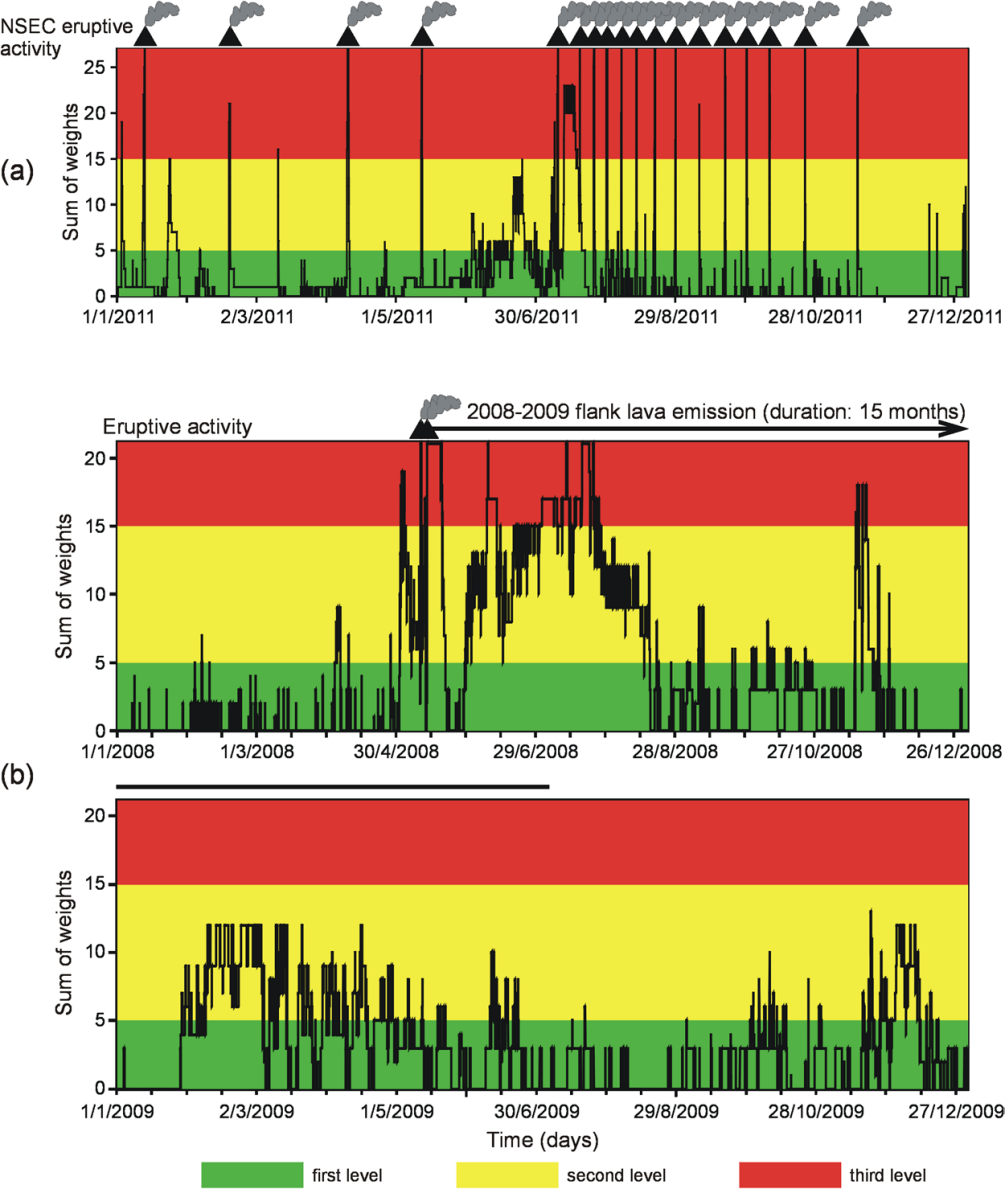
Application of the voting scheme to volcanic unrests at Etna in 2011 (**a**) and during the 2008–2009 flank eruption (**b**). Smoking volcano symbols mark the occurrence of lava fountains. Values of the sum of weights above 15 (red stripe) are associated with the climax of volcanic activity. The high values of the sum of weights achieved in Summer 2008 are concurrent with the uprising of new, fresh magma from depth documented by Corsaro and Miraglia^34^.

**Figure 6:**
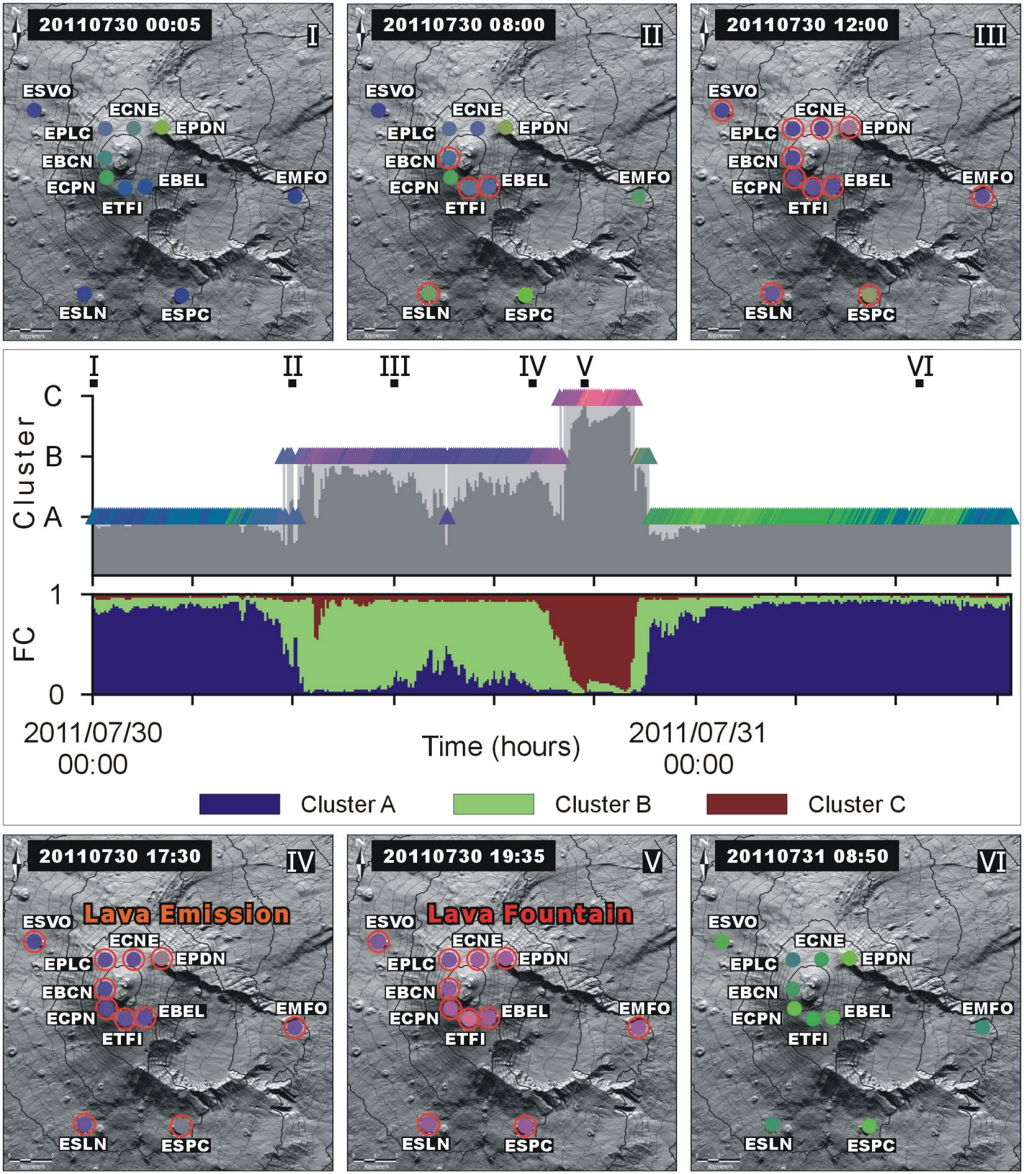
Pictorial representation of the SOM results (full circles) for each station on DEM; the six (from I to VI) successive time spans depict the color changes before, during, and after an episode of lava fountain on July 30, 2011. The border of a circle is highlighted in red in case of detection of a criticality. The shaded image of the DEM is from Favalli *et al*.^32^, reprinted by permission of the publisher (Taylor & Francis Ltd, http://www.tandfonline.com). Geographical coordinates as in Fig. 1. The diagram in the middle depicts the results at station EBEL over ~36 h. Results from SOM are given as a sequence of colored triangles. Concerning fuzzy clustering (FC), we report the prevailing cluster (A, B, C) and the fuzzy cluster membership values normalized between 0 and 1. The vertical position of the colored triangles in the SOM depends on the cluster to which a pattern prevalently belongs.

The system we designed is able to offer: (i) the automatic warning flag from a redundant number of stations, useful in case of temporary technical failure or noisy conditions at single sensors, and (ii) a good coverage of the summit craters potentially active. Unlike Boolean information – criticality yes/no – we set up a “House of stations” in which each seismic station could make an assessment. The simplest way to do it was just to consider “one station – one vote”. At this criterion we preferred a voting scheme with station weights (Fig. SI2). We assigned high weights to stations with optimal operational characteristics, such as reliability of acquisition and rare technical failures. Eventually, the vote resulting from the sum of weights was a value belonging to one of the three following score levels: 0–5 (green, no alert), 5–15 (yellow, pre alert), and >15 (red, alert) (Fig. 5). To get information on the occurrence of changes at each station and their spatio-temporal evolution, we also designed a pictorial representation of the SOM results on DEM. Figure 6 provides an example of such a visual, spatial depiction; the six successive time spans highlight the SOM color changes before, during, and after an episode of lava fountain on July 30, 2011. For the same eruptive episode, we provide a video containing the spatial depiction of the SOM with step of 5 min over ~36 h (video SI 1).

A description of the results of our voting scheme is reported in the Supplementary Information, considering two eruptive scenarios: the lava fountains in 2011 and the flank eruption in 2008–2009.

## Results

The development of systems for early warning is a complex, context-specific task, as every volcano has diverse hazards^23^. Any automatic warning system critically depends on both sensitivity and reliability. In volcano observatories, a sensitive system may detect even subtle criticalities before a volcano unrest occurs, but it would be at risk of generating “false” alerts, signaling criticalities when nothing is going to happen. Therefore, the identification of suitable criteria for the automatic flag requires the analysis of data warranting meaningful results in varying contexts, from gradual to sudden unrest/activity, despite noisy conditions due to, for example, bad weather^20^.

In the system presented here the presence or absence of a criticality is evaluated every time a new pattern is encountered. Given the specifications of data processing that means every 5 min. The sensitivity, i.e., the rate of flagged criticalities before an eruptive event, is controlled by the alert threshold. A low threshold may lead to numerous flags of “true positives”, i. e., criticalities coinciding with – even weak - volcanic activity, but comes with the cost of false alerts – “false positives”, i.e., flagged criticalities, which are not linked to the volcano but are caused by noise or signals not related to the activity of interest.

**Figure 7:**
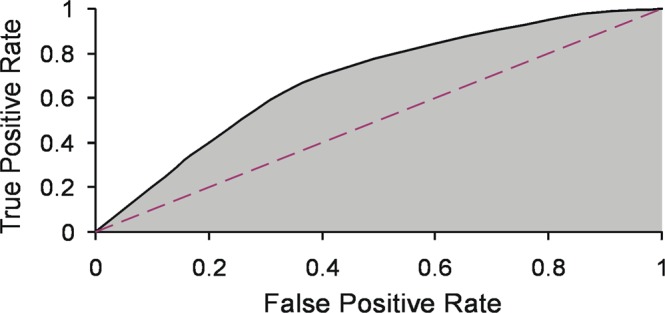
ROC curve choosing Strombolian activity as onset of the unrest (Table SI2). The Area Under the Curve (AUC; grey field) is ∼0.8. The diagonal of the curve (dashed line) marks the results of random behavior (AUC = 0.5).

In general, the efficiency of systems like ours can be tested comparing their performance with the results of a mere random choice by using so-called “Receiver Operating Characteristic” (ROC) curves^24^. ROC curves provide useful information on what kind of criticalities can be detected with a reasonable significance. The curves are obtained by plotting the “true positive rate” (TPR; ratio of the number of correctly flagged positives vs. the total number of true positives) against the “false positive rate” (FPR; ratio of the number of flagged false positives vs. the number of all true negatives, the latter being all observations where no volcanic activity occurred) at various threshold settings. Under pure random conditions, both TPR and FPR increase as the threshold lowers (purple dashed line in Fig. 7). Suppose we fix a high threshold. If the performance is good, we get generally high TPR, while FPR remains relatively low. Lowering the threshold, one gets both more “True” and “False Positives”, but the ROC curve remains above the diagonal shown in Fig. 7. A measure of goodness of performance is provided by the Area Under the Curve (AUC; see Methodology). As the use of ROC curves requires the a priori definition of a “true positive”, we referred to the volcanological observations collected by Behncke *et al*.^25^ as ground truth (Table SI2). The TPR was obtained from the number of flags falling in the time interval between “Start Strombolian Activity” and “End eruptive episode” (second and sixth column in Table SI2, respectively). Flags encountered outside this interval were considered as “false positives”. The columns “Start 2nd level warning” and “Start 3rd level warning” in Table SI2 report when the multi-station system switched from green to yellow and from yellow to red, respectively. The column “End 3rd level warning” reports the time when the system switched from red to yellow, closing the phase of maximum alert. In the following, we apply ROC as a tool to evaluate the global performance of the warning system based on the results of the voting scheme.

Etna has persistent activity, with continuous gas emission and sporadic explosions. Choosing Strombolian activity as onset of the unrest (Table SI2) makes the AUC ∼ 0.8 corresponding to a good performance (Fig. 7). Note that such an evaluation is a conservative judgement. Indeed, “false positives” do not occur randomly, but are typically before of or between the unrests reported in Table SI2. They may mirror a criticality in a very early stage, e. g., dynamics of a magma body, by no means revealed before. As we are interested in identifying criticalities well (hours) before the onset of fountaining and/or lava emission, we accept the chance of those false positives.

The numerous fountaining episodes in 2011 and the long-lasting 2008–2009 flank eruption (see details in Supplementary Information) formed a valuable set of volcano unrests to probe the multi-station warning system considering: (i) two different eruptive styles and scenarios, and (ii) the voting scheme in which each seismic station contributed according to its weight. In particular, the voting scheme allowed us to introduce a gradualness in the allocation of the results in one of three expected score levels (green, yellow, and red), depending on the sum of weights. The goals achieved implementing the multi-station scheme can be summarized as:

(i) gain in robustness – the warning system remains active even in case of instrumental failure of single stations
(ii) gain in sensitivity – the use of weights reduces false alarms
(iii) re-analysis of past data – offline analysis may reveal criticalities that escaped the attention of the operators or may have passed into oblivion
(iv) detection of the source of the unrest among various eruptive centers - the sensitivity of each station for the identification of criticality may vary according to the position of the station with respect to the eruptive crater/fracture.

The system applied the voting scheme to ∼2.7 × 10^6^ patterns of volcanic tremor.

A posteriori analysis of the results highlighted that some stations tend to signal more criticalities than others. In a few cases (e.g. ESPC), structural effects may play a key role^26^. Especially for the stations in the inner ring (e.g., ECNE and EBCN, Fig. 1), those warnings are likely caused by minor unrests of the volcano (e.g., sporadic Strombolian activity) or even failed eruptions, e.g. ascending magma batches not reaching the surface, but triggering gas pulses and rock fracturing. Such failed eruptions at Etna are associated with seismic unrest episodes recorded in the form of temporary enhancements of the volcanic tremor amplitude^27,28^.

In this work we processed seismic signals of the years 2008–2009 and 2011, considering a list of eruptive episodes for which the multi-station warning system would have signaled a criticality if it has already been running in the past. Its good performance and the affordable computational effort make it suitable for implementation in real-time monitoring and early warning purposes. Even though the system issues warnings based on pattern classification of volcanic tremor data, it can also process other kinds of data. The application of SOM to geochemical data have already provided promising results^15,27^.

### Methodology

SOM, or Kohonen maps^21^, are a particular family of neural networks consisting of a number of interconnected nodes. A node is a vector with the same dimensionality of input patterns. SOM are classifiers that “learn” from input data (vectors) and organize the information collected without the supervision of an expert. They are indeed based on suitable definitions of similarity between patterns rather than on a-priori knowledge of their class membership (no output/target vector has to be provided to the SOM). Similarity is defined on the basis of a distance measure. During the learning process, called “training”, the node weights are adjusted in order to minimize the sum of the differences between original data and their representing prototype nodes. Eventually, each SOM node represents a number of patterns; the topological relationship of the original data space is still maintained, i.e., patterns that are near in the original data space will be near in the SOM representation space.

In whatever 2D-graphical representation of SOM is used, the position of each pattern on the map mirrors in a RGB color code^29^. The definition of the color code is based on Principal Component Analysis, transforming the weights of the nodes in a 2D representation space made up of the principal axes z_1_ and z_2_. These axes are normalized in the range [0….1], becoming the red and the green component values, respectively. A third value, however, is necessary to obtain a valid RGB color code; z_3_ is then calculated either as 1–z_1_ or 1–z_2_ and used as blue component. The latter is only needed for the graphical representation, and brings no additional information, being complementary to one of the other two components. Each node receives a mixture of colors according to its original weight. By convention, in SOM with a ‘sheet’ geometry, a position at the upper-left corner of the map has a maximum saturation in blue. The green component increases from top to bottom, whereas blue decreases. Saturation in red augments moving from left to right. This kind of coding allowed us to visualize the development over time of the signal properties at each seismic station as a sequence of colored symbols.

Beside SOM, our multi-station system was also based on a simultaneous application of fuzzy cluster analysis^30^ according to which each pattern may belong - to a varying degree - to K possible classes. Therefore, the class membership of a pattern is given by a vector rather than being a simple identifier. The partition is consequently described by a M x K matrix of class membership values, with M being the number of patterns in the entire data set, and K the number of clusters. Following Langer *et al*.^20^, we fixed K = 3 classes (see clusters A, B, C in Fig. 6). This choice was guided by the aim of having as few clusters as possible, at the same time minimizing the degree of fuzziness. Applying fuzzy clustering, the class membership of a pattern is given by a vector normalized between 0 and 1 (FC values in Fig. 6); the partition is consequently described by a M × 3 matrix of class membership values.

In our application, the multi-station system compared the characteristics of volcanic tremor with those recorded under known scenarios of volcanic activity. This was obtained with the set-up of an input dataset consisting of two buffers. One buffer (the static one) was made up by a reference data set, which encompassed volcanic tremor data related to conditions of volcanic unrest in a few past eruptive episodes^20^. The second buffer (the dynamic ring) simulated real-time incoming data. It stored 24 hours of pre-processed signal from each single station, in a sliding window updated every 5 minutes. SOM and fuzzy clustering were carried out pooling data in the ring buffer together with the reference data set, which ideally should represent the parent population of possible scenarios of volcanic unrest. Note that whereas in Langer *et al*.^20^ there was just one reference data set (the classification involved one station only), we had reference data sets for each of the seismic stations considered.

The diagram in the middle of Fig. 6 depicts an example of changes in SOM color codes at station EBEL, along with the development of the fuzzy cluster membership, in six time spans encompassing an episode of lava fountaining. In the time span between I and II, the colors of the triangles are dominated by blue tones that are indicative of background conditions (quiescence). In the stage between II and IV (before the onset of the eruption), there is an increase in the red component; the latter prevails during the climax of the eruptive episodes (V). At the same time, the cluster membership changes from cluster A to B, and finally to C, the latter achieved during the climactic phase of the eruption. The return to the background conditions of the volcano (VI) is associated with cooler colors (green, blue) and the shift to cluster B and A. The pictorial representation of the SOM results on DEM in Fig. 6 highlights the development of the color changes in time and space at each seismic station.

### Receiver Operating Characteristics curves

In the set-up of the warning system, we had to consider two principal aspects which should govern its performance:

(i) High reliability in the detection of criticalities. In technical jargon, we ask for a high sensitivity, that is, the ratio of true positives (correctly detected positives – here criticalities linked to volcanic activity) with respect to all positives in our set of observations should be maximum (ideally 1 or 100%). We call it the “true positive rate” (TPR).
(ii) Limitation of non-existing criticalities, so-called false positives. As before, we define the “false positive rate” (FPR) considering the ratio of erroneously identified positives with respect to all negatives in the data set. Again, negatives are given by samples where no volcanic activity was observed.

In our application, the warning system was based on a threshold level, accounting for the number of stations and their individual weights. The definition of the threshold is a critical aspect, as it comes with the risk of issuing a warning even due to disturbances, their being noise or other signals not of interest here. There is an intrinsic trade-off between TPR and FPR that can be explored by using various thresholds. For this purpose, we created the “Receiver Operating Characteristics” (ROC) curves^24^ plotting TPR vs FPR for each threshold level. The performance of the warning system is given by the Area Under the Curve (AUC) which is a single number that summarizes the results obtained with all threshold settings (Fig. 7). There is a poor performance when TPR/FPR pairs follow more or less the diagonal of the curve. As a rule of thumb, a system “fails” if its performance is close to a random choice (0.5 < AUC < 0.6). The performance is “poor” for 0.6 < AUC < 0.7; “fair” for 0.7 < AUC < 0.8, “good” for 0.8 < AUC < 0.9, and eventually “excellent” for AUC > 0.9 (http://gim.unmc.edu/dxtests/roc3.htm; for more details on ROC and AUC, see e.g.^24,31)^.

A very sensitive system comes with the cost of a major number of false warnings. All entailed costs and consequences of the decision whether or not to issue a public warning require social and political evaluations which are beyond scientific tasks. Therefore, ROC curves are also useful for end users, such as governmental authorities or staff responsible for public communications, in making their decisions when the population may be at risk for the potential impact of volcanic activity.

## Supplementary information

Supplementary Information

Video
