# Supplementary material for: Short-term detection of volcanic unrest at Mt. Etna by means of a multi-station warning system
Source: Sci Rep. 2019 Apr 24;9:6506. doi: 10.1038/s41598-019-42930-3 (PMC6482307; doi:10.1038/s41598-019-42930-3)
Supplement: Supplementary file 1 — Supplementary Information [file 41598_2019_42930_MOESM1_ESM.pdf]

Supplementary Information for the article:

Short-term detection of volcanic unrest at Mt Etna by means of a multi-station warning system

Salvatore Spampinato[1], Horst Langer[1], Alfio Messina[2], Susanna Falsaperla[1]*

1. Istituto Nazionale di Geofisica e Vulcanologia, Sezione di Catania, Osservatorio Etneo, Piazza Roma 2, 95125, Catania, Italy

2. Istituto Nazionale di Geofisica e Vulcanologia, Sezione Roma2, Via di Vigna Murata 605, 00143, Roma, Italy

[*]Corresponding Author: susanna.falsaperla@ingv.it

This file contains:

- General information on volcanic tremor
- The 2011 case study
- The 2008-09 case study
- Figure SI1
- Figure SI2
- Table SI1
- Table SI2

General information on volcanic tremor

From the late 1970's, the seismic background radiation detected on active basaltic/andesitic volcanoes attained increasing attention. Schick and Riuscetti[1] documented at Etna clear relationships between variations in the spectral characteristics of tremor and changes to the state of the volcano. The characteristics of the signal, which is referred to as volcanic tremor, brought along the necessity of a continuous acquisition, requiring the storage of a large amount of data as well. Because long sequences of spectra (spectrograms) were cumbersome to handle, the application of pattern recognition techniques (Support Vector Machines, Multi-layer Perceptrons, Cluster Analysis, and Self-Organizing Maps – SOM hereafter) were explored[2,3,4]. In particular, Langer et al.[3] showed that at Etna: (i) regimes of volcanic activity mirror clearly in the spectral characteristics, and (ii) pattern recognition – in particular the SOM – allows a strong data reduction. Using SOM, the spectral characteristics of the signal are simply represented by a color code, which allows one to efficiently visualize the development of the signal properties over long time spans – just as a sequence of colored symbols. This simplification forms a bold step forward, as it allows the definition of straightforward criteria for early warning purposes.

In the following, we describe the outcomes of the voting scheme of our multi-station system considering two different eruptive scenarios: the lava fountains in 2011 and the flank eruption in 2008-2009. Overall, the system applied the voting scheme to ~2.7 x 10^6 patterns of volcanic tremor.

The 2011 case study

In-depth volcanological analyses document that all the eruptions in 2011 differed for durations, effusion rates, erupted volumes, and there was no correlation among the variations of these parameters[5]. Overall, there were 18 lava fountains. R and G components in the RGB color code of the SOM results highlight in Figure SI 1 the variations of volcanic tremor at the ECPN station shortly before, during, and after each eruptive episode. Numbered arrows mark the state of volcanic activity as documented by Behncke et al.[5] and reported in Table SI 2.

Figure 5a depicts the results obtained after applying the voting scheme to volcanic tremor data. The system issued a warning for each of the 18 episodes of lava fountains that occurred. During such short-lived (~2 h on average[5]) episodes, the sum of weights was above 15 (third score level, red stripe in Figure 5a), returning to lower score levels after the climax of eruptive activity (yellow and green stripes). Concurrent with Strombolian explosions in the period May-June 2011, the warning system signaled the shift to the second score level (yellow stripe; sum of weights between 5 and 15). With the resumption of lava fountains in July, the sum of weights reached again the third score level, peaking temporarily during each of the eruptive episodes (Figure 5a).

The 2008-09 case study

The second case study concerns the longest of the flank eruptions at Etna in this century. Volcanic activity was heralded by a lava fountain on 10 May 2008; lava flows started three days later and continued until 6 July, 2009[6]. Figure 5b depicts the results of the warning system throughout the whole period 2008-2009. In those years, the number of seismic stations was lower than in 2011, because a few stations were not yet installed. Accordingly, the voting scheme took into account 8 out of the 11 stations. The sum of weights was within the green stripe from January until the beginning of April 2008, and then it switched to yellow on 3 April, remaining at this level for a few days only. It returned to the yellow level again at the end of the month, concurrent with Strombolian activity between 23 and 28 April (Figure 5b). Even though the volcano was apparently quiet, the yellow level remained stable until 10 May when the warning system flagged the shift towards the red level, heralding the impending lava fountain. After a temporary return to quiescence, the volcano turned active again with lava flows on 13 May, shortly after a seismic swarm that opened the eruptive fractures. Afterwards, the eruption came to an abrupt halt, and no activity occurred over the following two days. Correspondingly (Figure 5b), the warning system switched to green and remained on this level until the beginning of June. A revival of both explosive and effusive activity started on 8 June, and continued unabated until the beginning of July. During this period, the sum of weights remained

between the yellow and red score level over weeks (Figure 5b), concurrent with the temporary arrival of fresh magma from depth documented by petrochemical analysis[6]. From the end of July on, explosive activity decreased whilst lava effusion continued; the score level changed from red to yellow and eventually to green. In the middle of November 2008, the warning system flagged again the red level. In this period, in-soil radon measurements peaked, reaching anomalous high values (~4.1 X 10^5 Bq/m^3)[7]. There was no other documented change, as neither video nor satellite imaging were available due to cloud cover. Bad weather conditions also hindered field surveys. In late 2008 and during the first months of 2009, the effusive activity continued at a score level that the system flagged yellow. Volcanic activity stopped and the warning system returned to the background level (green) in early July 2009.

References

1. Schick, R. & Riuscetti, M. An analysis of volcanic tremors at South Italian Volcanoes. *Zeitschr. Geophys.*, 247–262 (1973).
2. Masotti, M. et al. Application of Support Vector Machine to the classification of volcanic tremor at Etna, Italy. *Geophys. Res. Lett*. **33**, L20304, doi:10.1029/2006GL027441 (2006).
3. Langer, H. et al. Synopsis of supervised and unsupervised automatic classification techniques applied to volcanic tremor data at Mt Etna, Italy. *Geophys. J. Int*. **178**, 2 (2009).
4. Langer, H. et al. Detecting imminent eruptive activity at Mt Etna, Italy, in 2007–2008 through pattern classification of volcanic tremor data. *J. Volcanol. Geotherm. Res.* **200**, 1-17 (2011).
5. Behncke, B. et al. The 2011–2012 summit activity of Mount Etna: Birth, growth and products of the new SE crater. *J. Volcanol. Geotherm. Res.* **270**, 10–21 (2014).
6. Corsaro, R.A. & Miraglia, L. The transition from summit to flank activity at Mt. Etna, Sicily (Italy): Inferences from the petrology of products erupted in 2007–2009. *J. Volcanol. Geotherm. Res.* **275**, 51–60, doi:10.1016/j.jvolgeores.2014.02.009 (2014).

7. Falsaperla, S. et al. What happens to in-soil Radon activity during a long-lasting eruption? Insights from Etna by multidisciplinary data analysis. *Geochem. Geophys. Geosyst*. **18**, doi:10.1002/2017GC006825 (2017).

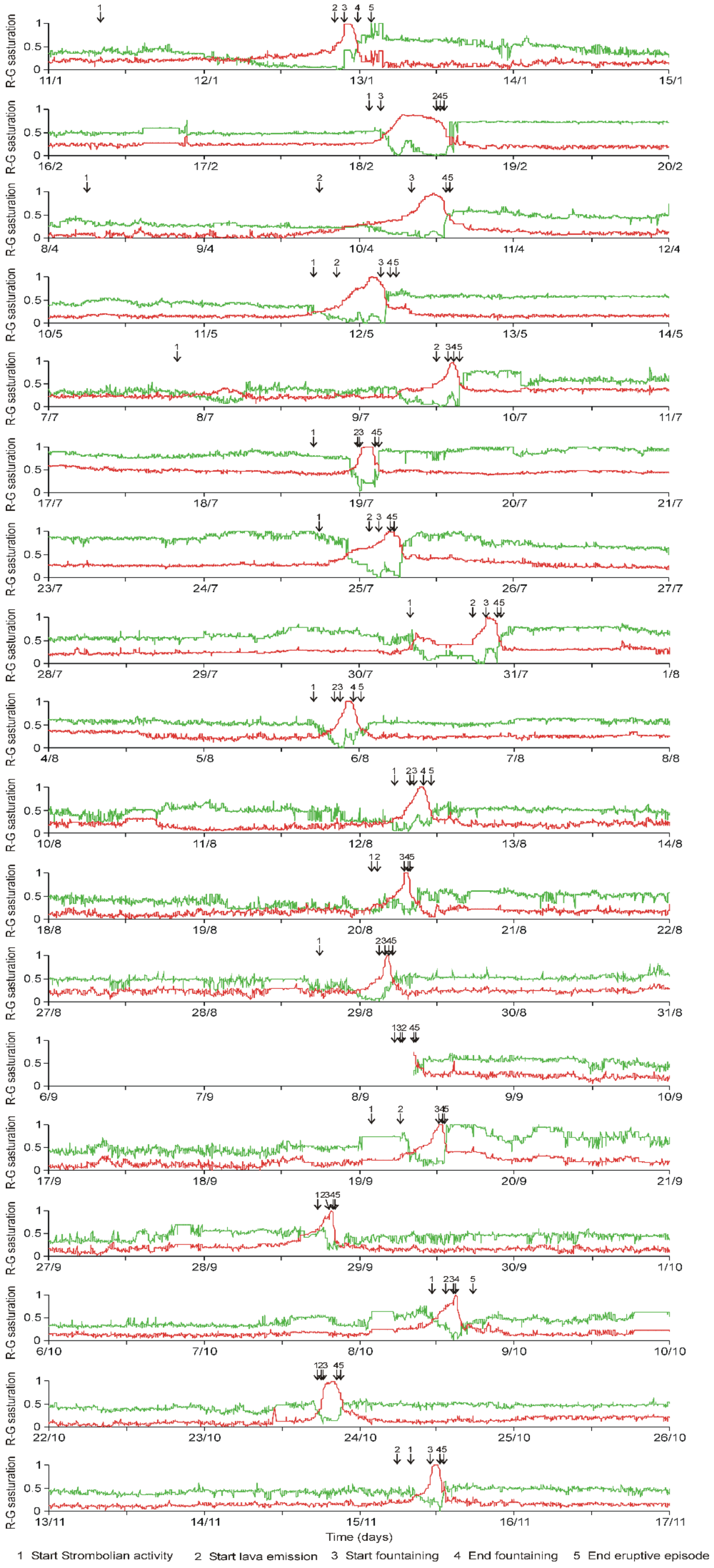
R-G sasturation
11/1 12/1 13/1 14/1 15/1
16/2 17/2 18/2 19/2 20/2
8/4 9/4 10/4 11/4 12/4
10/5 11/5 12/5 13/5 14/5
7/7 8/7 9/7 10/7 11/7
17/7 18/7 19/7 20/7 21/7
23/7 24/7 25/7 26/7 27/7
28/7 29/7 30/7 31/7 1/8
4/8 5/8 6/8 7/8 8/8
10/8 11/8 12/8 13/8 14/8
18/8 19/8 20/8 21/8 22/8
27/8 28/8 29/8 30/8 31/8
6/9 7/9 8/9 9/9 10/9
17/9 18/9 19/9 20/9 21/9
27/9 28/9 29/9 30/9 1/10
6/10 7/10 8/10 9/10 10/10
22/10 23/10 24/10 25/10 26/10
13/11 14/11 15/11 16/11 17/11
Time (days)
1 Start Strombolian activity
2 Start lava emission
3 Start fountaining
4 End fountaining
5 End eruptive episode

Figure SI 1. Normalized values of the red (R) and green (G) components in the RGB color code of the SOM results for the 18 episodes of lava fountains in 2011. The temporal variations of the R and G components refer to the ECPN station. Numbered arrows mark the state of volcanic activity as documented by Behncke et al.[5] and reported in Table SI 2.

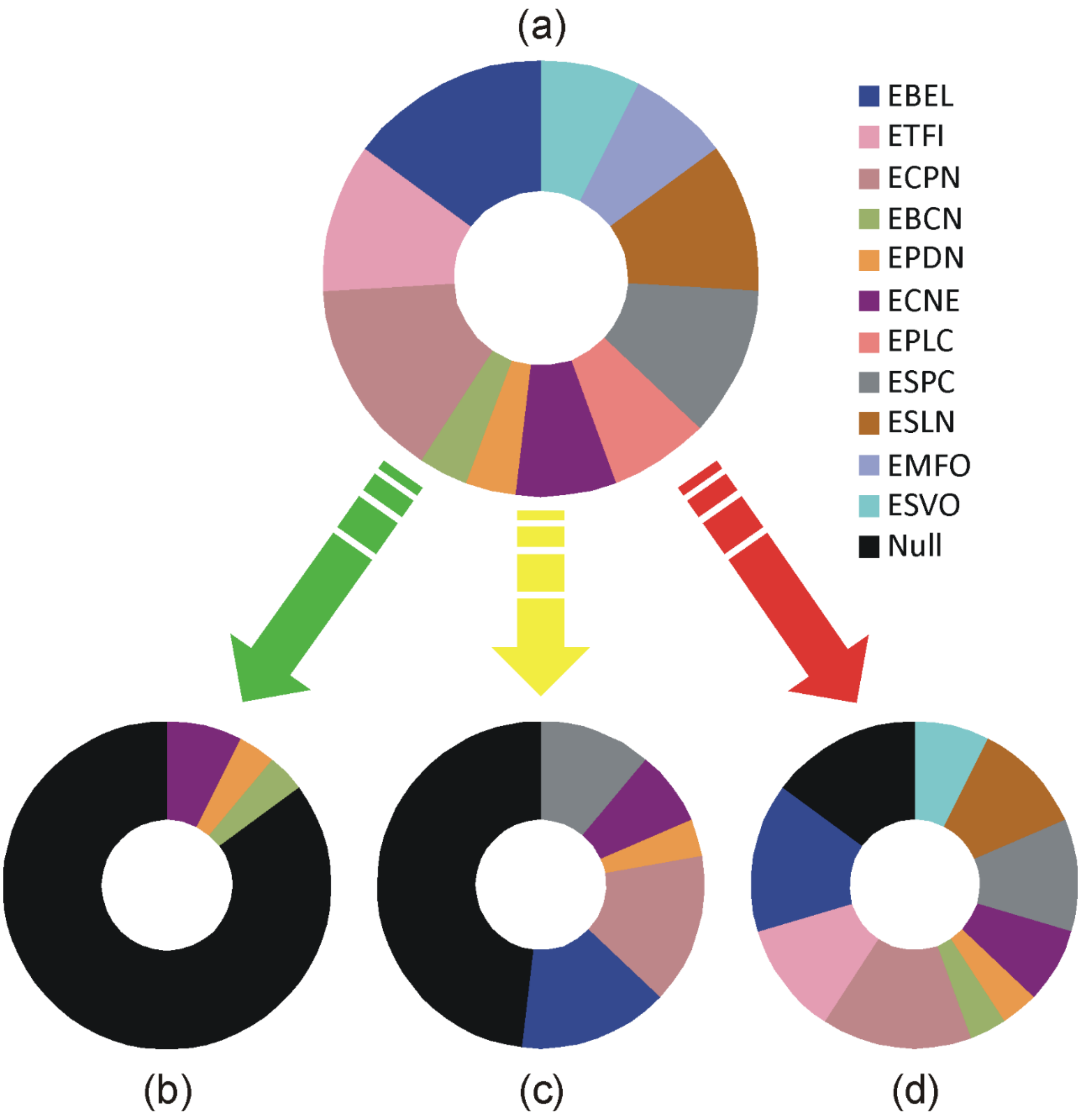

Figure SI 2. Sketch of the voting scheme for the alert system with weighted stations (a). Each station contributes to the final decision on the level to declare (green, yellow, and red arrows in the figure) according to its weight, here sketched by the size of its corresponding colored sector. When none or only a few stations pinpoint criticality, the “null” voters prevail (b, first score level). Increasing the number of stations that cast their vote for criticality, the level of warning may change to yellow (c) and eventually to red (d).

Table SI 1

Settings of trigger parameters for each single station of the warning system

Parameter / Station	EBCN	EBEL	ECNE	ECPN	EMFO	EPDN	EPLC	ESLN	ESPC	ESVO	ETFI
Number of consecutive "true" flags (**FlagsNum**) for alert starting or closing	7	7	7	7	7	7	7	5	7	7	7
Tollerance (%) of "false" flags in a FlagsNum sequence (**DiscToll**)	20	20	20	20	20	20	20	10	20	20	20
Threshold level for red saturation (upward) for alert starting (**MinRedLevelActiv**)	0.05	0.25	0.02	0.15	0.18	0.2	0.2	0.35	0.12	0.3	0.3
Threshold level for red - green difference for alert starting (**RedGreenOffsetActiv**)	-0.15	-0.25	-0.01	-0.05	-0.05	0.0	-0.05	-0.25	-0.1	-0.05	-0.25
Threshold level for green saturation (upward) for alert starting (**MinGreenLevelActiv**)	0.15	0.0	0.05	0.1	0.15	0.1	0.0	0.0	0.2	0.0	0.15
Minimum Cluster required for alert starting (**MinClusterActiv**)	A	A	A	A	A	A	A	A	A	A	A
Threshold level for red - green difference for alert closing (**RedGreenOffsetDisactiv**)	-0.15	0.0	-0.01	-0.05	-0.05	-0.05	-0.05	-0.25	-0.1	-0.05	-0.25
Threshold level for red saturation (downward) for alert closing (**MaxRedLevelDisactiv**)	0.4	0.25	0.4	0.4	0.3	0.4	0.4	0.4	0.4	0.45	0.4
Maximum Cluster allowed for alert ending (**MaxClusterDisactiv**)	B	B	B	B	B	B	B	B	B	B	B

Table SI 2

Eruptive episodes in 2011 and automatic warnings from the multi-station system

Episode	Start Strombolian activity	Start lava emission	Start fountaining	End fountaining	End eruptive episode	Start 2nd level warning	Start 3rd level warning	End 3rd level warning
01	Jan 11, 08:00	Jan 12, 20:10	Jan 12, 21:50	Jan 12, 23:50	Jan 13, 02:00	Jan 12, 00:10	Jan 12, 08:40	Jan 13, 00:35
02	Feb 18, 01:45	Feb 18, 12:00	Feb 18, 03:30	Feb 18, 12:30	Feb 18, 13:17	Feb 18, 03:35	Feb 18, 04:05	Feb 18, 15:25
03	Apr 8, 06:00	Apr 9, 17:55	Apr 10, 08:05	Apr 10, 13:30	Apr 10, 14:03	Apr 9, 20:30	Apr 9, 21:25	Apr 10, 15:25
04	May 11, 17:00	May 11, 20:30	May 12, 03:20	May 12, 05:00	May 12, 05:55	May 11, 17:10	May 11, 19:00	May 12, 05:55
05	Jul 7, 20:00	Jul 9, 12:05	Jul 9, 13:45	Jul 9, 14:45	Jul 9, 15:30	Jul 9, 06:00	Jul 9, 06:40	Jul 9, 17:00
06	Jul 18, 17:00	Jul 19, 00:00	Jul 19, 00:05	Jul 19, 02:30	Jul 19, 03:00	Jul 11, 23:05 (*)	Jul 18, 22:05	Jul 19, 03:50
07	Jul 24, 18:00	Jul 25, 01:30	Jul 25, 03:00	Jul 25, 05:00	Jul 25, 05:30	Jul 24, 20:55	Jul 24, 22:10	Jul 25, 08:35
08	Jul 30, 07:50	Jul 30, 17:30	Jul 30, 19:35	Jul 30, 21:30	Jul 30, 22:00	Jul 30, 07:35	Jul 30, 08:30	Jul 30, 22:45
09	Aug 5, 17:00	Aug 5, 20:15	Aug 5, 21:00	Aug 5, 23:00	Aug 6, 00:15	Aug 5, 18:35	Aug 5, 19:05	Aug 6, 01:55
10	Aug 12, 05:30	Aug 12, 07:50	Aug 12, 08:30	Aug 12, 10:00	Aug 12, 11:00	Aug 12, 03:55	Aug 12, 05:25	Aug 12, 12:25
11	Aug 20, 02:00	Aug 20, 02:55	Aug 20, 07:00	Aug 20, 07:30	Aug 20, 07:50	Aug 20, 00:00	Aug 20, 02:15	Aug 20, 10:05
12	Aug 28, 18:00	Aug 29, 03:15	Aug 29, 04:05	Aug 29, 04:40	Aug 29, 05:15	Aug 28, 23:35	Aug 29, 01:10	Aug 29, 06:45
13	Sep 8, 05:30	Sep 8, 06:50	Sep 8, 06:30	Sep 8, 08:30	Sep 8, 08:45	------	Sep 8, 08:35 (°)	Sep 8, 10:30
14	Sep 19, 02:00	Sep 19, 06:30	Sep 19, 12:20	Sep 19, 13:00	Sep 19, 13:10	Sep 19, 07:10	Sep 19, 08:00	Sep 19, 16:30
15	Sep 28, 17:30	Sep 28, 19:15	Sep 28, 19:31	Sep 28, 19:55	Sep 28, 20:10	Sep 28, 14:45	Sep 28, 15:45	Sep 28, 21:40
16	Oct 8, 11:24	Oct 8, 13:30	Oct 8, 14:30	Oct 8, 14:50	Oct 8, 17:45	Oct 8, 11:10	Oct 8, 11:35	Oct 8, 18:05
17	Oct 23, 17:40	Oct 23, 18:07	Oct 23, 18:30	Oct 23, 20:30	Oct 23, 21:15	Oct 23, 17:20	Oct 23, 18:00	Oct 23, 22:10
18	Nov 15, 08:00	Nov 15, 06:00	Nov 15, 11:00	Nov 15, 12:29	Nov 15, 13:00	Nov 15, 08:35	Nov 15, 08:40	Nov 15, 13:50

Data from the 2nd to 6th columns are from Behncke et al.[5]. All times are UT.

(*) From this time until 22:00 on July 18, the warning system flagged numerous criticalities, and the level flipped several times before steadily reaching the third level related to the fountaining episode.
(°) For this episode the warning system issued a delayed alert due to a temporary crash in the seismic data acquisition until 08:30 on September 8.
